# Adipocytokines in Rheumatoid Arthritis: The Hidden Link between Inflammation and Cardiometabolic Comorbidities

**DOI:** 10.1155/2018/8410182

**Published:** 2018-11-21

**Authors:** Piero Ruscitti, Paola Di Benedetto, Onorina Berardicurti, Vasiliki Liakouli, Francesco Carubbi, Paola Cipriani, Roberto Giacomelli

**Affiliations:** Rheumatology Unit, Department of Biotechnological and Applied Clinical Sciences, University of L'Aquila, Delta 6 Building, L'Aquila, PO Box 67100, Italy

## Abstract

Rheumatoid arthritis is a chronic autoimmune disease affecting typically synovial joints and leading to progressive articular damage, disability, and reduced quality of life. Despite better recent therapeutic strategies improving long-term outcomes, RA is associated with a high rate of comorbidities, infections, malignancies, and cardiovascular disease (CVD). Remarkably, some well-known pathogenic proinflammatory mediators in RA, such as interleukin-1*β* (IL-1*β*) and tumor necrosis factor (TNF), may play a pivotal role in the development of CVD. Interestingly, different preclinical and clinical studies have suggested that biologic agents commonly used to treat RA patients may be effective in improving CVD. In this context, the contribution of adipocytokines has been suggested. Adipocytokines are pleiotropic molecules, mainly released by white adipose tissue and immune cells. Adipocytokines modulate the function of different tissues and cells, and in addition to energy homeostasis and metabolism, amplify inflammation, immune response, and tissue damage. Adipocytokines may contribute to the proinflammatory state in RA patients and development of bone damage. Furthermore, they could be associated with the occurrence of CVD. In this study, we reviewed available evidence about adipocytokines in RA, because of their involvement in disease activity, associated CVD, and possible biomarkers of prognosis and treatment outcome and because of their potential as a possible new therapeutic target.

## 1. Introduction

Rheumatoid arthritis is a chronic autoimmune disease affecting typically synovial joints and leading to progressive articular damage, disability, and reduced quality of life [1–4]. RA is associated with an increased rate of comorbidities, including infections, malignancies, and cardiovascular disease (CVD), leading to the excess of mortality experienced by these patients [5–7]. Remarkably, a close association between RA and accelerated atherosclerosis has been highlighted, due to the interaction between traditional cardiovascular (CV) risk factors and proinflammatory pathways [8–11]. Furthermore, the fact that traditional CV risk factors are underdiagnosed and undertreated may increase the atherosclerotic process [12, 13]. In addition, some well-known pathogenic proinflammatory mediators in RA, such as interleukin-1*β* (IL-1*β*) and tumor necrosis factor (TNF), may play a pivotal role in the development of CVD [14–16]. In fact, common pathogenic inflammatory pathways between the atherosclerotic process and rheumatic diseases have been shown [16–18]. Different reports have suggested that biologic DMARDs, commonly used to treat RA patients, may be effective in improving CV comorbidities [19, 20]. In this context, the contribution of adipocytokines has been suggested [21]. Adipocytokines are pleiotropic molecules, mainly released by white adipose tissue and by immune cells [21, 22]. Adipocytokines modulate the function of different tissues and cells, amplifying inflammation, immune response, and tissue damage [21]. During RA, adipocytokines could contribute to the proinflammatory state, develop bone damage, and accelerate concomitant atherosclerosis [22–25].

In this study, we reviewed available evidence about adipocytokines in RA, because of their involvement in disease activity, associated CVD, and possible biomarkers of prognosis and treatment outcome and because of their potential as possible new therapeutic targets.

## 2. Methods

We designed a narrative review aimed at providing an overview about leptin, adiponectin, resistin, and visfatin in RA, because of their involvement in disease activity, associated cardiometabolic diseases, and possible biomarkers of prognosis and treatment outcome and because of their potential as possible new therapeutic targets. We performed an analysis of available evidence linking the same molecule to joint damage and cardiometabolic comorbidities, in order to discuss previous studies but also to provide a rationale for further researches. MEDLINE (via PubMed) was searched and the bibliography of relevant articles was also hand searched for identification of other potentially suitable studies.

## 3. Adipocytokines in RA: Generality, Pathogenic Mechanisms, and Changing Pattern to Treatment

### 3.1. Leptin

Leptin is a 16 kDa nonglycosylated adipocytokine with a long-helix structure and it is one of the most common adipocyte-derived molecules [26]. Leptin shows different biological actions deriving from an activation of OB-Rb long-form isoform receptors, which are encoded by the diabetes (db) gene [27]. Acting on hypothalamic nuclei, leptin decreases food intake and increases energy consumption, via induction of anorexigenic factors and suppression of orexigenic neuropeptides [28]. Furthermore, leptin is involved in both innate and adaptive immune responses being its production influenced by proinflammatory mediators [21–23]. Specifically, this adipocytokine exerts proinflammatory activities upregulating the production of TNF, IL-6, IL-1*β*, and IL-12, which, in turn, increase the expression of leptin in adipose tissue [21, 28, 29]. Leptin modulates the activity of innate immune cells by (i) enhancing the phagocytic activity of monocytes/macrophages; (ii) stimulating chemotaxis and release of reactive oxygen species by neutrophils; and (iii) promoting NK cell differentiation, proliferation, activation, and cytotoxicity [27, 30–32]. Concerning the effects on adaptive immune cells, leptin is able to (i) stimulate proliferation of naïve T lymphocytes and activate B cells; (ii) shift the T-cell cytokine production towards a Th1 phenotype, increasing the production of IFN-*γ* and IL-2; and (iii) induce regulatory T-cell anergy and T-cell receptor-reduced responsiveness [33–35]. As shown by a recent meta-analysis, circulating leptin levels are significantly higher in RA patients compared with controls [36]. Furthermore, it has been reported that obese RA patients showed an increased production of leptin according to ACPA positivity, thus suggesting that leptin could favour the humoral response against citrullinated proteins [37]. In addition, Olam et al. assessed the ratio between serum leptin levels and the synovial fluid [38]. Synovial/serum leptin ratio was significantly higher in RA patients and correlated with disease duration, disease activity, proinflammatory cytokines, and acute phase reactants [38]. However, conflicting results are also available in the literature and future studies are needed to elucidate the pathogenic role of leptin in RA [39, 40]. In fact, although this adipocytokine is considered to be proinflammatory, it has also been reported to be associated with reduced radiographic joint damage and this effect could be related to the anabolic effects of leptin [39, 40].

Recently, many studies assessed the effects of biologic DMARDs on leptin in RA, considering a relevant issue the changing pattern of this molecule after treatments [41–43]. RA patients treated by TNFi were investigated for leptin levels, assessing serum levels before and after such treatment [42, 43]. Interestingly, leptin levels did not change, suggesting that the beneficial effect of TNFi therapy on CVD outcomes in RA could not be mediated by a reduction of leptin [44, 45]. In fact, no significant modification was observed assessing leptin levels during therapy with adalimumab, etanercept, and infliximab [41–45]. However, these studies should be cautiously interpreted because the number of enrolled patients was relatively small.

### 3.2. Adiponectin

Adiponectin is a 244-residue protein, also known as GBP28, apM1, Acrp30, or AdipoQ, and it is mainly synthesised by adipose tissue [46]. This adipocytokine increases fatty acid oxidation and glucose uptake in the muscle and reduces the synthesis of glucose in the liver, acting via 2 receptors, AdipoR1 and AdipoR2, found in skeletal muscle and liver, respectively [47]. Ablation of the adiponectin gene has a dramatic effect in knockout mice on a high-fat/high-sucrose diet, inducing insulin resistance and lipid accumulation in muscles [46, 47]. Mirroring animal models, adiponectin levels are lower in obese patients and higher in patients losing weight [48, 49]. On the contrary, adiponectin and its receptors increase during physical activities [50]. Furthermore, the secretion of adiponectin is inhibited by proinflammatory cytokines, suggesting that inflammation may contribute to hypoadiponectinemia in insulin resistance and obesity [51].

In rheumatic diseases, adiponectin could act as a proinflammatory mediator in joints and it could be involved in matrix degradation [52, 53]. During RA, adiponectin and AdipoR1 expressions were higher in the synovial fluids and synovial tissues of patients compared with those of controls [54]. In this study, many cells derived from RA synovial fluids and tissues, including synovial fibroblasts, showed adiponectin, adipoR1, and adipoR2. Interestingly, the addition of adiponectin to cultures of synovial fibroblasts increased the production of proinflammatory cytokines, such as IL-6 and IL-8 [54]. The stimulation with adiponectin also contributed to the production of metalloproteinases, such as MMP-1 and MMP-13, by RA synovial fibroblasts [55]. Furthermore, adiponectin could synergise with IL-1*β* thus increasing the production of proinflammatory mediators by RA synovial fibroblasts [56, 57]. Adiponectin aggravated bone erosions by promoting osteopontin production in RA synovial tissue, suggesting that adiponectin induced the expression of osteopontin, which in turn recruited osteoclasts [58]. Recently, the effects of adiponectin were assessed on adipose mesenchymal stem cells (ASCs) derived from the infrapatellar fat pad of RA patients [59]. ASCs were stimulated with both low molecular weight (LMW) and high/middle molecular weight (HMW/MMW) adiponectin isoforms. The authors observed that the secretion of proinflammatory mediators was upregulated by HMW/MMW adiponectin, but not by LMW adiponectin. In addition, they observed that the stimulation with HMW/MMW adiponectin reduced the proproliferative effects of ASC-derived soluble factors on RA synovial fibroblasts [59]. Taking together these results, it is possible to suggest a proinflammatory and joint destructive role of adiponectin in RA [55–59].

### 3.3. Visfatin

Visfatin is a protein of 471 amino acids and 52 kDa, and it is produced by the liver, bone marrow, muscle, macrophages, and visceral adipose tissue [60, 61]. This adipocytokine is increased in obesity [61]. Visfatin is regulated by proinflammatory cytokines and, in turn, it induces chemotaxis and the production of inflammatory cytokines, such as IL-1*β*, IL-6, and TNF, in lymphocytes from obese patients, suggesting involvement in the obesity proinflammatory *milieu* [62]. Furthermore, the proinflammatory actions of visfatin have been observed in experimental models of arthritis, in which the high levels of visfatin were proposed to modulate the proinflammatory process and the joint destruction [63, 64].

During RA, serum visfatin levels were higher in patients and correlated with radiographic joint damage [65–67]. Despite the association with radiographic outcome, the correlation with disease activity has shown conflicting results. In fact, the association with disease activity reported in some studies has been not confirmed in others [66–68]. The relatively small sample size and different experimental conditions could partially explain these results. Similarly, the analysis of results derived from clinical studies evaluating the changing pattern of visfatin after treatment with TNFi showed conflicting results. Serum visfatin levels were analysed in RA patients, who were differently treated (i) after 16 weeks of adalimumab treatment, (ii) after 2 weeks of high-dose prednisolone, and (iii) after 22 weeks of treatment with a combination regimen with tapered high-dose prednisolone and synthetic DMARD. Treatment with adalimumab was associated with a reduction in visfatin levels, whereas in other groups of patients, opposing effects on visfatin levels were observed [42]. On the contrary, other authors showed that visfatin levels did not change after the administration of infliximab [68].

### 3.4. Resistin

Resistin is a 12.5 kDa protein included in the resistin-like molecule (RELM) family, and it is mainly produced by nonadipocyte resident inflammatory cells, mainly macrophages [69–71]. Resistin increases with obesity and promotes insulin resistance, suggesting a possible link between obesity and diabetes [72–74].

Although a significant difference was not found in serum resistin levels between patients and controls, a pathogenic role for resistin has been suggested in RA [75, 76]. In fact, the intra-articular injection of recombinant resistin in the knee joints of murine models induced arthritis and increased the production of several proinflammatory cytokines, such as an increased expression of several proinflammatory cytokines including IL-1*β*, IL-6, IL-12, and TNF [76]. Furthermore, higher levels of this adipocytokine were observed in synovial fluid samples from RA patients and were correlated with disease activity and joint damage [77]. These data could suggest the production and the contribution of resistin in the inflamed joint, despite the lack of correlation with inflammatory markers in peripheral blood [76, 77].

Concerning the changing pattern after treatment, TNFi reduced serum resistin levels [42, 78]. After the administration of infliximab, the serum resistin levels significantly decreased in RA patients. In this cohort, resistin levels also correlated with inflammatory markers thus suggesting a possible role in the RA inflammatory process [78].

## 4. Adipocytokines and Cardiometabolic Diseases in RA

**Figure 1:**
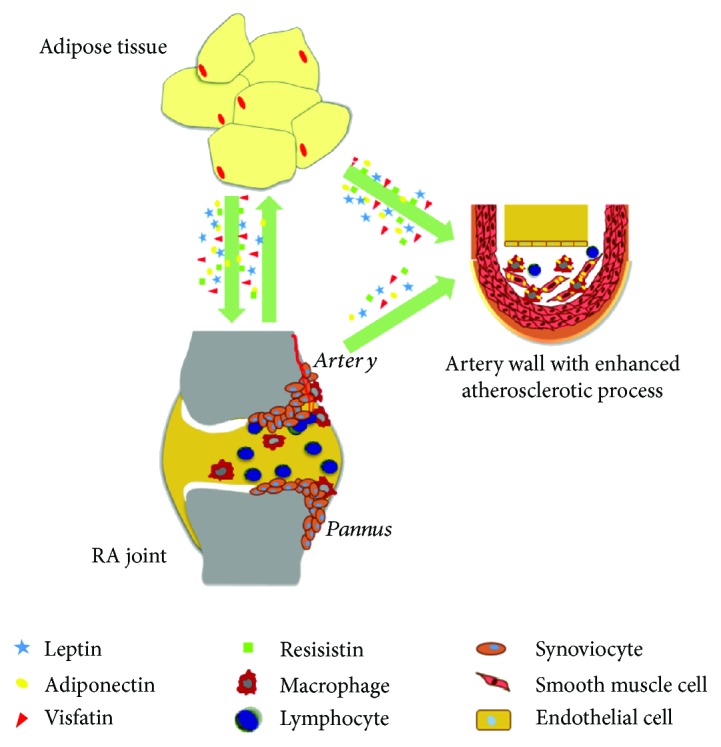
Schematic role of adipocytokines on the relationship among adipose tissue, rheumatoid arthritis, and atherosclerotic process.

**Figure 2:**
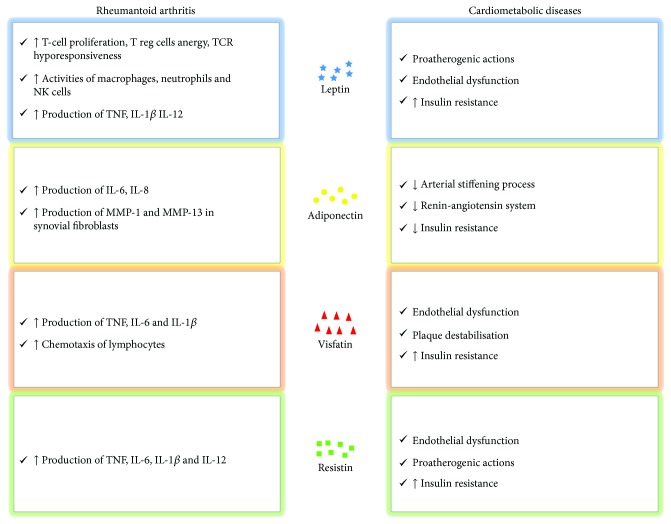
Pathogenic mechanisms of adipocytokines in rheumatoid arthritis and cardiometabolic diseases. Abbreviations: T Reg cells—T regulatory cells; TCR—T-cell receptor; NK cells—natural killer cells; TNF—tumor necrosis factor; IL—interleukin; MMP—metalloproteinase.

RA patients characteristically experience an increased risk of CVD derived from the synergy between traditional CV risk factors and inflammation [7–10]. In this context, the role of adipocytokines has been suggested as a possible link between adiposity, inflammation, and cardiometabolic diseases (Figure 1) [79, 80]. A previous study was performed to evaluate whether adipocytokines could affect insulin resistance and coronary atherosclerosis in RA patients [81]. In this study, the authors assessed the coronary calcium score, homeostatic model assessment for insulin resistance (HOMA-IR), and serum adipocytokine (leptin, adiponectin, resistin, and visfatin) levels in 169 RA patients. To date, high leptin levels correlated with HOMA-IR, even after adjustment for possible clinical confounders, age, gender, BMI, traditional CV risk factors, and inflammatory mediators. On the contrary, visfatin, adiponectin, and resistin showed no association with the HOMA-IR index. No association was retrieved between the coronary calcium score and assessed adipocytokines [81]. More recently, adipocytokines were further investigated as a link between inflammation, insulin resistance, and atherosclerosis in RA, being associated with pathogenic mechanisms of these diseases (Figure 2) [82]. A study evaluated HOMA-IR, intima-media thickness (IMT), carotid artery (CCA) resistive index (RI), and carotid plaques in 192 RA patients. These data were correlated with levels of adiponectin, leptin, and resistin. The authors observed that leptin and leptin : adiponectin (L : A) ratio were correlated with HOMA-IR and with CCA-RI after adjustment for CV risk factors, suggesting a possible independent role of leptin in predicting CVD in RA [82]. Although these correlations were not observed in another experience [83], it is possible to speculate that leptin is associated with insulin resistance in RA. Multiple lines of evidence showed the influence of leptin in the metabolism of glucose and pathogenesis of insulin resistance and diabetes [84, 85]. Insulin resistance in diabetic leptin receptor-deficient or genetic leptin-deficient animal models could not be fully attributed for their obesity and hyperphagia; the restriction in caloric intake failed to improve or recover the sensitivity of insulin in these models [86]. Furthermore, leptin administration in these models reduced plasma insulin and blood glucose levels [87]. In addition, leptin could influence glucose metabolism via the modulation of glucagon by *α*-cells of pancreas [88]. Furthermore, leptin could provide a functional link between obesity and CVD [88]. The link between fat mass and atherogenesis is confirmed by the findings in animal models of obesity [89, 90]. Leptin levels were associated with endothelial dysfunction proatherogenic actions, enhancing oxidative stress in endothelial cells, smooth muscle cell proliferation, and vascular calcification [90].

Concerning adiponectin, the correlation between total and HMW adiponectin concentrations, cardiometabolic risk, and surrogate markers of enhanced early atherogenesis was performed in 210 RA patients [91]. Total and HMW adiponectin concentrations correlated with high systolic, diastolic, and mean blood pressure and HDL cholesterol concentrations, low total HDL cholesterol ratios and triglyceride concentrations, and triglyceride-HDL cholesterol ratios and glucose concentrations [91]. These results mirrored what was observed in a lipoatrophy mouse model with adiponectin deficiency [92]. In these models, the replacement of adiponectin improved insulin resistance, fatty acid oxidation, and energy consumption, leading to a reduction of triglyceride levels in muscle and liver tissue [92, 93]. Furthermore, wild-type mice which received a high-fat diet showed a reduction in adiponectin levels and the replacement of adiponectin improved this diet-induced hypertriglyceridemia [94, 95]. To date, the possible role of adiponectin in modulating the homeostasis of blood pressure has been suggested [96]. In a cross-sectional study assessing patients with high blood pressure, high serum adiponectin levels were correlated with low procollagen type I carboxy-terminal propeptide circulating levels, a molecule reported to be associated with the arterial stiffening process [97]. Furthermore, adiponectin showed the ability to increase the gene expression and to activate the endothelial nitric oxide synthase by activation of AMPK [98]. Finally, it has been reported that adiponectin inhibited the deleterious effect of the renin-angiotensin system on the vascular system [99].

The potential impact of visfatin was assessed on CVD in 232 RA patients [100]. Visfatin concentrations were related to increased diastolic blood pressure and presence of diabetes [100]. In this context, it has been reported that visfatin could represent a proinflammatory cytokine influenced by insulin and/or insulin sensitivity via the NF-*κ*B and JNK pathways [101, 102]. The role of visfatin was investigated in the impairment of the insulin pathway by TNF activity in adipocytes. In that study, the authors showed that visfatin was involved in TNF-mediated insulin resistance in adipocytes, via the NAD(+)/Sirt1/PTP1B pathway [103]. Furthermore, heterozygous mice with a mutation in the visfatin gene had higher levels of plasma glucose, impaired glucose tolerance, and reduced glucose-stimulated insulin secretion when compared with controls [104]. In addition, high visfatin levels could mediate vascular damage by inducing the expression of adhesion molecules via oxidative stress-dependent NF-*κ*B activation, thus leading to endothelial inflammation and plaque destabilisation [105]. However, conflicting results are available concerning the role of visfatin [106, 107], thus future studies are needed to entirely clarify its role in cardiometabolic diseases.

Finally, the role of resistin has been proposed in cardiometabolic diseases. Of note, a certain degree of crosstalk between resistin and other adipokines has been reported [108, 109]. In fact, the expression on endothelial cells of VCAM-1 and ICAM-1 by resistin is counteracted by adiponectin [108]. A further link between leptin and resistin has also been proposed, and the expression of resistin was shown to be suppressed by leptin administration in animal models with subsequently decreased glucose and insulin levels [109]. In addition, the pathogenic role of resistin in atherogenesis has been proposed [110]. The secretion of resistin from atheroma-derived macrophages was suggested because of the colocalization of resistin and CD68 in the staining of human aneurysms and the higher mRNA resistin expression in cultured macrophages than in controls [111].

## 5. Adipocytokines as Future Possible Therapeutic Targets

In the last decades, long-term outcomes of RA have remarkably improved by using synthetic and biological DMARDs [112–114] and, presently, multiple lines of evidence assessed the best therapeutic strategy of concomitant diseases [115, 116]. In this context, it has been proposed that the inhibition of some cytokines may extend beyond the inflamed joints thus targeting, at the same time, associated comorbidities and improving the management of these patients [115–117]. Taking together these observations, it could be possible to speculate whether targeting adipocytokines may be effective in RA and comorbidities. Presently, antagonists of leptin have been developed to treat metabolic disorders. It should be tested if they could also have anti-inflammatory activities *in vivo* [118–120]. Interestingly, a monoclonal antibody against the leptin receptor was shown to block human TNF production by monocytes acting as an antagonist [121]. Recently, an orally active adiponectin receptor agonist improved insulin resistance and glucose intolerance in mice [122]. Considering that adiponectin showed anti-inflammatory properties, it could be speculated that adiponectin or adiponectin receptor agonists could be promising targets for the development of therapeutic drugs to treat insulin-resistant states and possible inflammatory states [123].

## 6. Conclusions

RA is a chronic autoimmune disease with increased mortality, due mainly to CVD. Adipocytokines are shown to be of importance in the pathogenesis of RA and associated comorbidities. Future studies are needed to identify the new mechanisms of action of adipocytokines and to elucidate if these molecules could be new possible therapeutic targets, thus improving the management of RA patients.
